# Social Networks, New Technologies, and Wellbeing—An Interview Study on Factors Influencing Older Adults’ Successful Ageing

**DOI:** 10.3390/ijerph20075279

**Published:** 2023-03-27

**Authors:** Alina Betlej

**Affiliations:** Centre of Sociological Research on the Economy and the Internet, The Department of Economic and Digital Sociology, The John Paul II Catholic University of Lublin, Al. Racławickie 14, 20-950 Lublin, Poland; alina.betlej@kul.pl

**Keywords:** successful ageing, wellbeing, new technologies, social networks, ageing

## Abstract

Many factors are considered vital in supporting successful ageing and older adults’ wellbeing. Whilst evidence exists around facilitating and hindering factors in the general use of various forms of institutional and family support and personal development-oriented education and/or new technologies, evidence is limited with regards to older people’s motivations, expectations, and experiences surrounding ageing. Hence, in this study, the author used a qualitative explanatory method to interpret the factors influencing seniors’ successful ageing. The author’s focus was on how seniors experience ageing. The second issue was how they have been organizing life in old age. The third point concerned their expectations towards ageing now and in the future. Thirteen older adults (60+) were interviewed nationwide using a semi-structured scenario tool. Their objective was to give rich descriptions of their experiences of ageing. The interviews revealed the older adults’ own experiences and enabled an understanding of their motivations, perceptions, moderators, and expectations around successful ageing. Based on the analysis of the qualitative data, the author developed three main themes, each with its own sub-themes: 1. Life satisfaction (transitioning to retirement, using coping strategies in adaptation to negative changes, reaching personal goals, leading a meaningful life); 2. Supportive environments (being independent but using temporary assistance from relatives and/or people close to oneself, living with family members (e.g., husband or wife, children, grandchildren), having access to health care system); 3. Social integration (social relations, social engagement, independence in using technological advancements). The main categories that emerged from the three themes were social networks, new technologies, and wellbeing. To analyze these issues, the author used a sociological approach. The theoretic explorations were embedded mainly in two methods: criticism of writing and the analytical and comparative one.

## 1. Introduction

Over the past decade, many theories have been proposed to describe successful ageing [1,2]. Central to most of these conceptualizations has been the development of universal definitions of success, ranging from non-involvement to longevity. However, ongoing research has not led to a single general theory of successful ageing. It remains a major challenge to identify criteria or even interpretive schemes for success in old age that could be universally accepted by representatives of the social sciences. Currently, there is a visible trend towards a return to research on successful ageing [3,4]. The reasons may be found in specific social and economic transformations. The analysis of statistical data confirms that intense demographic ageing of societies around the world is a modern phenomenon [5]. Never before has there been observed by demographers such a shift in the distribution of a country’s population towards older ages as manifested in an increase in the mean and median age of the residents. Nowadays, the trend is imbalanced in proportion to people’s median ages, with a decrease in the population of children and a rise in the population of older adults. Declining fertility, increasing longevity and the transition of large cohorts into old age are resulting in a growing percentage of seniors in societies worldwide.

The phenomenon of population ageing is unprecedented in human history. Implications of these transformations are severe in many domains of social activity e.g., meeting the needs of older people, their capabilities and potentials, health care system changes, consumptions changes, asset values and fiscal sustainability, social responsibility, ethical issues etc. Despite knowledge of these demographic changes, unflattering stereotypes about older people still occur in various commonalities. It should be noted that more and more attention is now being drawn to the lives and daily activities of older people [6]. A number of factors are considered to be central to successful ageing and wellbeing in older people [7]. While there is evidence of facilitators and barriers to the widespread use of various forms of institutional and family support, personal development-oriented education and/or new technologies, there is limited evidence regarding older people’s motivations, expectations, and experiences of ageing [7]. It is an important task to conceptualize the concept of successful ageing on the basis of a literature review and the qualitative research conducted by the author, with a focus on how older people experience ageing, the organization of life in old age and their expectations towards ageing now and in the future.

The paper is structured as follows: Section 2 reviews the literature on conceptualizations of successful ageing. Section 3 develops the methodology and Section 4 describes the research findings. The discussion in Section 5 gives answers to the research question in the literature context of past allegations. Section 6 reflects on the study’s limitations and suggests avenues for future research. Finally, Section 7 indicates the study’s conclusions.

## 2. Literature Review

The notion of success has different connotations today. It is a social construct that embraces diverse meanings. Understandings of success has evolved throughout history. In the past, success has been associated with having good luck or bad fortune as well as with value judgements like positive outcome or any outcome. In contemporary findings, success is linked to beneficial assets deriving from an individual’s activity [7]. Success is often measured in terms of economic accomplishments [8]. Contemporary understandings of the term go beyond the financial outcomes to refer to more complex issues [9]. Success can refer to the achievement of all kinds of personal goals. Examples may include keeping good health, generativity, optimizing physical activity, self-realization, social relations, continuing personal career, being in a relationship etc. [10].

Conceptualization of successful ageing brings researchers’ attention to many varied theories and approaches. Although some claims could be made that this notion is an oxymoron and/or related to a capitalistic point of view, it is still a very interesting topic on social analyses because of the fact that we are living in an era of ageing societies around the world. Internationally, it is recognized that ageing can be an affected, complicated, and sometimes difficult process for individuals adapting to a new social situation [11,12,13]. Although influenced by the social and cultural context of the given times, we may trace a deep ambivalence about ageing in well-known theories. With the advent of modern societies based on economic values of productivity, knowledge, and creativity, we observe an interesting shift in how successful ageing has been analyzed. Seniors are no longer perceived through the prism of their productivity. Certain stereotypes about ageing are still very powerful nowadays, for example, discriminatory and exclusionist views of seniors. The scientific theories of the process may be considered as interesting frames for studying this phenomenon.

The scientific attention to ageing may be analyzed by theories that facilitate a more negative or evaluative view on the process by focusing almost solely on seniors’ problems, e.g., their isolation, dependence on others, role-loss, identity crises, illness and/or other health issues [14,15]. In the last decade, we have witnessed essential changes in understanding ageing as a social phenomenon. Nevertheless, there has been a noticeable trend in the development of gerontological theories that focus on the decline or growth of ageing populations [16]. Interdisciplinary research findings, especially in biological, psychological, and sociological backgrounds, have proven the potential of older individuals. We find examples in many domains. A cognitive ageing perspective explains the importance of improved learning and performance conditions [17]. Social behaviour orientation analysis analyzes the transition from dependent to self-dependent care activity when constructed proper social backgrounds to give needed prompts [18]. The huge amount of physical inheritance can also be contrasted with corresponding attitudes to wealth. Biological scientists have described physical health, longevity, and functional autonomy as vital for successful ageing for many years [2,13]. Possible losses in old age are understood as ambivalent factors that may be influenced by specific and hidden potentials of seniors.

Theories of successful ageing may be divided into two categories. The early approaches place a strong emphasis on the adaptive effects of old age. In doing so, they are able to overcome some of the limitations of gender and focus attention instead on humanity and wisdom. These factors have been identified as facilitators of successful ageing [18]. Psychological points of view have focused on criteria of the ageing process such as psychological peace and ego integrity of individuals [18]. In addition, many newer approaches have considered the acceptance of decline as the assignment of adaptation in old age [19]. These analytical perspectives may also include the disengagement theory which emphasized that success should be analyzed through the prism of acceptance and reconciliation, as well as the loss of power endemic in old age [20]. In contrast, activity maintenance and the replacement of lost roles with new ones have been defined as mediating factors in successful ageing in activity theory [21,22]. This focus on social engagement and the importance of social relations has paved the way for further research on the formula for successful ageing [15]. One effect of such thinking is the development of an integrative model of the ageing process based on developmental, clinical, and mental health approaches [23]. It has become clear that many aspects of life should be considered when assessing successful ageing. The mentioned integrative model includes six dimensions: self-acceptance, positive relations with others, autonomy, environmental mastery, purpose in life, and personal growth [18]. In this analytical stream, successful ageing is inextricably linked to a sense of meaning and purpose in life, and therefore refers to paradigms or existential ideals [24].

More recent avenues emphasize that ageing may be considered through the prism of the radicalization of the individual condition, and thus it is often described in opposition to the two statements of self-actualization and self-alienation [25]. Psychological variables such as experienced social support and lifestyle appear to influence health status and even mortality. Interestingly, good physical health is not a main determinant in these approaches. According to this approach, the criteria of functional autonomy and longevity do not necessarily lead to psychological wellbeing [26,27]. In previous studies, successful ageing was described by way of a formula of satisfaction with life, high morale, and subjective assessment of wellbeing [1,2,3]. Life satisfaction was often analyzed by researchers as a universal index of success [27]. A sociological perspective on old age primarily focuses on issues such as the characteristics of the older generation, the place of the old in society, the place of the old in the family, the place of the old in the labour market, the social expectations of seniors, loneliness and solitude and their conditions, the withdrawal process from previous social roles, the values defined and accepted by the older generation, the social roles played by older people, and ways of living in care homes [28,29,30]. Thus, sociological theories place more emphasis on social structural variables and influences on life satisfaction, for example, income/financial situation, population density, marital status, level of education, and institutional backgrounds for ageing society [31]. Importantly, not only the objective indicators have an impact on successful ageing. Sociological thinking leads us to consider the population of old people as a generation, as a social category, and as a socio-professional group [6]. A sociological view of successful ageing can be defined as analyzing old people through the prism of their social roles and their activities in groups, collectives, and social structures [6]. Sociologists are interested in the problem of compensating for roles lost in the course of ageing, mainly professional and social roles, as well as the traditional role of family provider. Thus, successful ageing is studied in frames of social exclusion theories with a special emphasis on digital exclusion and inclusion [31,32].

It should be noted that the contemporary research also pays significant attention to loneliness, which is operationalized as living alone, being alone, and the level of social isolation and health degradation, as a prevalent phenomenon within ageing populations [33]. Previous literature has suggested that social technology use may alleviate loneliness and can be defined as an important factor of successful ageing in modern societies. Another point of contemporary sociological research is to reveal mediating indicators of particular strategies of organizing life in old age [6]. In line with this research perspective are studies that consider the experiences of people entering care homes [3,34,35]. Some research studies explore the experience of losing autonomy, independency, and identity as challenging factors during old peoples’ adaptation to a new social situation.

This paper addresses the gaps in the literature by exploring individual and cultural ways of understanding successful ageing as they are embedded in specific cultural environments and from the perspective of older people. According to this paper’s author, we need to rethink the concept of successful ageing and the notion of success in old age. Most theories of ageing do not consider the individual, social, or cultural variability of older people’s goals and expectations. Typically, similar and/or universal clichés are used to perceive and evaluate this complex process. Many potentially competing axionormative orders coexist in social reality. Norms drawn from one culture are not always helpful in analyzing social problems and complex social situations characteristic of another cultural background.

## 3. Materials and Methods

### 3.1. The Design/Methodology/Approach

This study on older adults’ experiences of successful ageing uses participating persons’ narratives as empirical data to explore the most important factors mediating people’s motivations, assessments, and expectations surrounding ageing. The collection of primary data and the study is based on a qualitative research method. A storytelling practice was combined with the scenario of a semi-structured interview to stimulate discussion about individuals’ perceptions, thoughts, and feelings about ageing. Its purpose was to give rich descriptions of selected problems, e.g., how older adults experience successful ageing, organize life in old age, and what expectations they have of ageing. Narratives construct ideas, allowing for a real engagement in the evolving dialogue with participants about their lives and their plans to change these [36,37]. Importantly, the researcher makes an assumption about the process of the social construction of reality. This approach is reflected in the chosen research methodology. The type of interviews conducted and the way in which the collected research data is analysed allows for an understanding of selected social, economic, and, above all, cultural phenomena. Based on this way of thinking, the researcher rejects the scheme of causal interpretation of events. In the course of the analysis, he introduces and develops idealistic ontological solutions, especially notions of human experience and expectations. The procedure involves more in-depth descriptions and analysis, such as the “how” and “why” [38,39]. The findings are interpreted with reference to the chosen theories. The author developed the open-ended scenario of the interview to embrace emerging circumstances and discoveries during the research. The researcher also permitted the form and content of the scenario to change during the interviews to capture as much of the narratives as possible. The interview questions were designed based on older adults’ experiences of ageing. Three main stages were classified as the most important: distinct spans of life, diverse activity arenas, and the participants’ peculiar interactions with other people. These stages relate to the essential elements of narrative research, namely temporality, spatiality, and sociality, and translate into the core category of the data analysis.

To this end, the author articulated the following leading questions, which were developed into more specific questions during the research:

1. Tell me more about your life before retirement?
2. Tell me about how you are getting since you are retired? (perception of self-worth, wellbeing, and health condition)
3. Tell me how has your life changed since stopping professional work? How do you feel about these changes in your life?
4. Tell me about any concerns and/or worries/fears you may have about ageing now?
5. Tell me about your knowledge/competences of using new technologies?
  ⋅ Do you use social media? Is it easy/difficult? If not, why?
  ⋅ Do you use a smartphone? Is it easy/difficult? If not, why?
  ⋅ Do you use a computer? Is it easy/difficult? If not, why?
  ⋅ Do you use Skype, Teams, Zoom? Is it easy/difficult?
  ⋅ Do you use any age-friendly assistive technologies? Is it easy/difficult? If not, why?
6. Tell me about how you keep in touch with family and friends? If not, why?
7. Tell me about other ways that you keep in touch with others? If not, why?
  ⋅ Do you participate in social groups? (Seniors clubs, third age universities, hobbies groups, local social organizations, parish charities groups, support groups etc.; why, where, for how long, for what reason?)
  ⋅ Do you usually meet people face to face/personally? (why, where, for how long, for what reason?)
8. Tell me if you would like to change something in your life right now, thinking about your future? If not, why?
9. What do you think the future holds?

### 3.2. Participants/Sampling

The sample of 13 older people was recruited across Poland in 2022 following the purposive sampling strategy of maximum variation [39], which amounted to “identifying differential characteristics or criteria for constructing the sample” [39]. The recruited participants were from the following areas, providing a range of rural and urban locations: the Lublin Province, the Subcarpathia Province, the Lodz Province, and Mazowieckie. Each person was over the age of 65, lived in Poland, and had either experienced transitioning to retirement in the 12 months prior to the start of the study or would retire within that time. In this research, the identified varied characteristics were age, gender, and education level. The researcher also applied a snowball sampling to search for seniors fitting the approved methodology, which meant that the participants may help in recruiting future subjects from their acquaintances.

The ranging age of the participants was expected to be from 65 to 80. The author was looking for individuals with different education levels as well as various work experiences, as both were defined as important mediating factors in the study. 13 participants were interviewed during the study, of which 5 were male and 8 female. Participation was voluntary with no expectations for compensation. Author informed the participants about the aim of the study, the research data management procedure, and about anonymity. The interviewees were also assured of their right to refuse to participate in or to withdraw from the study at any stage. They all agreed to participate in the interviews. The interviewees were identified by codes in the study (Table 1). The study protocol was approved by the Research Ethics Committee of the John Paul II Catholic University of Lublin.

The definition of levels of education can vary greatly across different countries, which may lead to problems with international comparisons of statistics. The International Standard Classification of Education (ISCED) is the official framework used to facilitate international comparisons of education systems based on more general classifications such as early childhood education, primary education, lower secondary education, upper secondary education, post-secondary non-tertiary education, short-cycle tertiary education, bachelor’s degree or equivalent tertiary education level, masters degree or equivalent tertiary education level, and doctoral degree or equivalent tertiary education level. The interview participants referred in their answers mainly to the following three general stages of education: primary = equivalent to the completion of 8 grades of primary school; secondary = completion of secondary school (general secondary school or technical secondary school); higher = completion of higher education (masters degree).

The older adults were asked how they felt about their health (for their age).

The theoretical sampling/data saturation was the second sampling strategy applied in the study [40]. This procedure is based on the assumption that the coding process itself determines the validity of interviewing a certain number of participants. During it, new concepts, categories, and relationships between them emerge. The next stage of the procedure is to make comparisons between the emerging narratives of everyday life. Once theoretical saturation has been reached, the research procedure should be terminated, as it can be concluded that no new analytical categories will be discovered in the subsequent interviews. According to these two sampling strategies, the researcher continued interviews until no new concepts emerged through continuing sampling, and the implementation of new interviews would not significantly help in solving the research problem [41,42].

### 3.3. Data Collection

Data were collected through in-depth open-ended interviews conducted in Poland in 2022 and early 2023; the interviews lasted from 1.5 to 2 h. The study was guided by a pilot study of 4 people aged 70+ in October 2022 that aimed to evaluate the study protocol and a research scenario. The interview scenario allowed for ongoing interpretation of the narratives of the seniors and some conceptual agreement. The data collection tool was an in-depth semi-structured interview. The in-depth strategy facilitated the development of new thematic threads that emerged during the conversation, which were discussed and used to establish new meanings [41,42]. In the case of the semi-structured interviews, the same questions were asked in each interview. However, in every case the seniors had the option of adding new, important conceptual avenues related to the interview following the storytelling practice to give extensive narratives.

The interviews were audio-recorded on a dictaphone and transcribed verbatim. The transcription process took between 6 and 7 h for each interview; after that procedure the transcripts were then analyzed by the researcher. A second procedure of accuracy in categorizing the concepts was provided by double-checking each code. Transcription accuracy was re-checked at the end of the study to correct any transcription errors. Coding reliability was approved by a cross-coding analysis of the coded data. During the current data analysis, around 90% of all codes were identified by the eleventh interview. This is defined in terms of data saturation, i.e., a situation in which organizing more interviews would not contribute significantly to solving the research problem [41,42]. The author assumed that the sampling procedure was methodologically correct and that the number of participants was not a limitation of the research.

### 3.4. Data Analysis

The research material collected was very extensive. The interviews were relatively long. In them, the seniors talked about many aspects of their lives. The author was therefore able to explore their individual motivations, their perception of the reality around them and their own barriers to ageing, as well as many other subjective experiences of old age. The author therefore had to read a large number of transcripts and focus on looking for similarities and/or differences between them whilst finding common themes in order to then create appropriate categories. The researcher applied the above-mentioned three dimensions of experiencing ageing (past, present), organizing life in old age (specific social situations and strategies of sociality), and expectations of successful ageing (future perspective). This analytical logic thus unfolded along a specific time horizon of past-present-future. Hence, the author’s analytical procedures were embedded in a qualitative, explanatory method to understand factors influencing individuals’ successful ageing. The author’s focus was on how they were experiencing ageing. The second issue was how they have been organizing life in old age. The third point concerned their expectations towards ageing now and in the future. The author interviewed 13 older adults (60+) in Poland.

The author read the interview transcripts many times. Her intention was to understand the world as it is experienced by people and saturated with values. The researcher was therefore a reflective participant in each interview. The next step in the methodological procedure was the evaluation of the research data, which was carried out using the coding approach chosen by the author. This refers to conceptual abstraction, i.e., the assignment of generic concepts (codes) to individual events recorded in the data. The researcher used three types of coding procedures, namely open coding, axial coding, and selective coding. Performing the coding opened the way for further analysis of the research material. During open coding, the researcher conceptualised and categorised events. She then proceeded with axial coding to explore the relationships between concepts and categories. In the final stage, the author carried out selective coding and combined the different categories into a coherent theory. The result of these procedures are the categorised statements of the seniors. The data were then assigned to the categories of identified themes collected in the study (see Table 2). The interviews and analysis of the transcripts were conducted in the Polish language and then translated into English by the researcher.

## 4. Results

In total, the coding process revealed over 160 concepts, of which 130 relevant ones were applied creating a total of three categories (see Table 2). The number of concepts in each category ranged between 13 and 25. These categories covered: (1) Life satisfaction—(a) transitioning to retirement, (b) using coping strategies in adaptation to negative changes, (c) reaching personal goals, (d) leading a meaningful life; (2) Supportive environments—(a) being independent but using temporary assistance from relatives and/or people close to oneself, (b) living with family members (e.g., husband/wife, children, grandchildren), (c) having access to health care system; and (3) Social integration—(a) social relations, (b) social engagement, (c) independence in using technological advancements. Together, these three categories shape the foundations of the core category “social networks, new technologies, and wellbeing”. The emerged themes and subthemes are described in the following section. The author has added to each category the quotes of the seniors to illustrate their various experiences.

### 4.1. Life Satisfaction

#### 4.1.1. Transitioning to Retirement

Transitioning to retirement was described by the participants as crucial for the future self-evaluation of successful ageing. The turning point was the crossing between the two worlds of employability and the search for a new idea for oneself.

*I had been saying all my life that I couldn’t wait to retire, to finally relax and get on with my business. And when the time was right, I felt a kind of desperation that was hard to explain. I felt that I was crossing some kind of boundary line beyond which nothing was waiting for me. At that point I didn’t want to retire yet. I felt that this was my best time for professional development.*

PLM88

One of the participants did not face this problem of transitioning to retirement while ageing. In the context of difficult life changes, she claimed that all depends on the nature of professional work and family circumstances. She perceived the passing of time through the growing up of her children.

*I treat ageing as something natural. I have always had this attitude. Previously, I worked as a farmer and took care of the house, the family, and the children. A difficult experience for me was when my children moved away from home, first to go to university and then to start their own lives. I was still young then, but it was a difficult time for me. I don’t have such specific memories related to just getting older, that from the next day onwards I already belong to older people because I’m retiring, for example. For me it was such a smooth, natural process.*

PLF77

One of the participants pointed to an interesting issue of different expectations towards ageing.

*I was looking forward to retirement. I remember that in the first week after retirement, I rested. In the second week, I started to do various overdue repair work on the house. After a month, I fixed everything, cleaned up everything and thought to myself, ok I’ve rested, and I’ve caught up on various household chores and what’s next? What am I going to do? I was already starting to get bored in this retirement [enough].*

PLM73

Transitioning to retirement was also seen as a moment of life breakdown and the new beginning of successful life.

*I think I needed a break from professional work at the time, a change of environment. I was already bored with certain things for so many years. I needed a new challenge. I think I made good use of that moment of transition.*

PLM81

#### 4.1.2. Using Coping Strategies in Adaptation to Negative Changes

The participants mentioned the importance of using specific coping strategies to adapt to new and/or difficult situations. One of the participants claimed that these competences improved while ageing.

*It seems to me that successful ageing depends on how we know how to deal with difficult situations. And I think this skill comes with age.*

PLF77

One of the most important coping strategies was searching for new activities and setting yourself new tasks and goals to be more motivated to take up new actions.

*I was feeling a kind of emptiness at the time, I didn’t know how to fill this free time. I mobilised myself to find some new interesting activity.*

PLF72

One of the participants said that while good health was not the only predictor of successful ageing, it was important to develop a mechanism for dealing with situations of poor physical and mental condition.

*As long as we don’t have any health problems everything goes on somehow. The hardest thing is to face a sudden illness or some limitations that we haven’t experienced before… My family helped me a lot then, but I also wasn’t ashamed to talk about it and ask for help.*

PLM73

Moreover, being independent in life at its previous stages meant not asking for help.

*Sometimes it’s hard, I can’t say there I haven’t difficult moments in my life… I’ve always had a problem asking others for help.*

PLM88

#### 4.1.3. Reaching Personal Goals

The assessment of successful ageing was often discussed by the participants in the context of their past lives. One of the mediating factors was reaching personal goals defined differently.

*I always had a lot of plans, but I still didn’t have enough of anything.*

PLM81

One of the interviewees regretted not being able to realize all his life plans. These kinds of feelings and experiences influenced ways of thinking about success in a few cases.

*I didn’t achieve everything and I feel a bit sorry for myself now…. I could have behaved differently in some situations.*

PLM88

Success in life itself was also understood very differently during the interviews. Some referred to the concept in terms of professional success, while others referred to private success.

*Success is such a relative term. It means something different to everyone. For me, the most important thing was to live in harmony with myself, with what my parents taught me, to be good to people.*

PLF72

*I had a good husband, wonderful children. Now I am happy with my grandchildren. For me, family was always the most important thing. Other things were not so important. It’s known that money comes and goes, so there’s no need to focus on it.*

PLF77

When reflecting on their success, participants often compared themselves with others. Many life situations were referred to in the context of being in interdependence with others.

*Sometimes I find myself comparing myself to others and I have this feeling of slight deficiency that I could have achieved more in life.*

PLM73

#### 4.1.4. Leading a Meaningful Life

The interviewees illustrated the things, experiences, and events that constituted a meaningful life. One of them was focused on having a respected social position thanks to their chosen professional work.

*I definitely have that sense of satisfaction that what I did in life was important to others. I have always been a respected person, people counted on my opinion.*

PLM88

Others claimed that negating the importance of money and committing instead to the family, love, and the beauty of the everyday were the core categories of success.

*I am now very proud of my children’s achievements. I can see that the time and attention given to them was important. I am really proud that my husband and I were able to raise such wonderful and smart children.*

PLF77

One of the participants who devoted his life to his professional career and forgot many times about his family regretted that strategy.

*I used to devote a lot of time to my career, even at the expense of my family. I always thought that what I was doing at work was so important. Now I regret that a little bit. I see that it’s all so fleeting. At work, everyone has long forgotten my merits. Younger people have come in and are doing things their own way.*

PLM73

Success was also linked to getting a dream job that afforded independence and freedom of self-expression.

*I remember when I started my career, I dreamt of being independent at work. I didn’t want to be just another part of the system. I wanted to dress my own way and be able to organise my working time individually. I started working under communism and it was not at all easy. After a dozen years or so, I managed to get such a job and it has a real meaning for me.*

PLM81

### 4.2. Supportive Environments

#### 4.2.1. Being Independent but Using Temporary Assistance from Relatives and/or People Close to Oneself

The participants linked successful ageing with being independent in daily activities and accepting help from people close to oneself such as family members, friends, and close ones. The interviewees pointed to living in nursing homes as the main threat to successful ageing.

*I think successful ageing will end for me when I am no longer independent and end up in a care home. How much easier it would be for me to accept the help of family and loved ones in such a situation, I can’t even imagine that I could end up in such an institution.*

PLF72

The participants often preferred living alone but also admitted having problems coping well with daily duties.

*I would like to continue living alone in my own home, but I know that time is passing and I am no longer coping well with everything. I hope that in the future my children will be helping me.*

PLF77

Living in a nursing home aroused the participants’ associations with losing independence.

*I am afraid that I might end up in a care home for the elderly in the future. I am afraid that I would be treated badly there. I have always been independent in my life, I rarely ask others for help, so this would be difficult for me.*

PLM88

One of the participants said that receiving help from people close to her did not impose the feeling of being dependent on others.

*My children live far away from me. My neighbours help me on a daily basis, and sometimes I ask for help from extended family. This help gives me a sense of independence, that I somehow manage my daily chores. I don’t have a driving licence, so the hardest things for me are shopping, going to the doctor. I can no longer carry heavy things.*

PLF65

#### 4.2.2. Living with Family Members (e.g., Husband/Wife, Children, Grandchildren)

Many participants related successful ageing with living with their family members.

*My health is very bad, but I live with my family, who support me in everything, and somehow I put up with it. I wouldn’t want them to put me in a care home.*

PLM73

*I envy those who live with their children.*

PLM88

One of the interviewees perceived living with the family as the predictor evoking a feeling of being important for someone.

*I live with my daughter, her husband and grandchildren and I am very happy about that. I don’t feel lonely and I can count on their help every day. I also help them and it makes me feel important.*

PLF70

The participants also had future plans related to moving into their children’s homes.

*For now, I live alone but I would like to live with my son’s family in the future when they can no longer cope with everything or I become ill. I certainly wouldn’t want to live in an old people’s home.*

PLM81

#### 4.2.3. Having Access to Healthcare System

The participants associated ageing with health problems now or in the future and saw having access to a good healthcare system as a necessity. The limitations were linked to money to pay for private services. In other situations, the distance to a health center and good family doctor were pointed as important for people in old age.

*I certainly think more about my health now. I would like to have access to a good doctor. Everyone says it’s best to use private doctors, especially dentists. For that you need money.*

PLF72

*I now appreciate that I live so close to the health centre. It’s a very big value. I don’t have to ask anyone to drive me.*

PLM88

*In older age, it is certainly important to have access to good healthcare and a good family doctor.*

PLM81

One of the participants perceived potential medical issues through the prism of their difficult experiences with close friends who were dying.

*Many of my friends have died of cancer. I have seen their suffering. I am afraid that someday it may happen that my doctor will not diagnose me properly.*

PLF70

Some participants in the study had a generally bad opinion of the health service in Poland.

*As they say in our country, to heal means to have health [laughs]. The health service in Poland is a failure. You can wait up to two years to see a specialist. If you want to be examined quickly, you have to have money to have a private visit to a doctor.*

PLM73

### 4.3. Social Integration

#### 4.3.1. Social Relations

All respondents highlighted the importance of social relationships in their personal lives. They interpreted loneliness as the greatest threat in old age. Seniors pointed to the meaningfulness of being in some interrelation with others, with the specific focus of family and or people close to them.

*Believe me, social isolation and the feeling of being alone is one of the biggest problems of people who experience ageing. You even can’t imagine how it is to not have anyone to say hello to him hello during the day if you have not experienced lioness.*

PLF72

>*Successful ageing is for me living in some relationships with other people. If you experience lioness it means you have failed.*

PLM88

The participants defined being in some relation to others as crucial for wellbeing. They considered a life of solitude and lack of contact with other people as a condition for a dignified existence.

*I have always looked for connections, relations, and meeting new people. I know I can’t live without others around me.*

PLF70

Relationships were often reduced to the category of contact with others, not always based on strong bonds and regular interactions.

*Well, maybe the people I meet now are not always the ones to be called friends but it’s enough for me to have colleagues or just people which I meet at the church, shop or on the road to the shop to whom I can talk, ask about how they are doing and have a conversation.*

PLM81

*Unless there is a possibility of some relations with others, I feel happy and I hope that future brings me many such relations and interesting people on my way.*

PLF76

#### 4.3.2. Social Engagement

The interviewees mentioned specific forms of social involvement such as participating in senior citizens’ clubs, universities of the third age, rural housewives’ circles, and church organisations in local parishes.

*I can say that I’m actively ageing [laugh]. I don’t sit at home and despair. I’m active in the Senior Citizens’ Club, I’ve been to lectures at the University of the Third Age. And I’ve actually only now had the opportunity to visit half of Poland thanks to my involvement in the Senior Citizens’ Club. I didn’t have time for that before. It was work, it was the children, and there was no time for myself.*

PLF77

*I’m constantly involved in something, whether it’s social action or the University of the Third Age. I travel around Poland giving lectures, and I meet new people all the time.*

PLF76

Social involvement was associated by them not only with self-development but also with continuing professional activity.

*I am a member of the church group meeting in my parish…but I use here my professional knowledge and experience. I really put a lot of effort into this.*

PLF72

*I am a member of the rural women’s club. I am very much involved in our work. I can use my experiences here to teach the youngest how to cool local cuisine…We also support others, such as our local firefighters. We organise joint events…*

PLF70

#### 4.3.3. Independence in Using Technological Advancements

Independence in using technological advancements was perceived by the participants as a very important factor of successful ageing. Moreover, the level of digital competences was evaluated as a predictor determining a number of social relations in a closer and wider social environment. The seniors claimed themselves to be users of digital devices and social media many times. They described themselves as not being ‘technology minded,’ and in these cases the estimated level of independence in using digital devices was related with perceived self-confidence and self-efficacy, which directly impacted not only online social relations but also social engagement in offline activities.

*To be honest, I started my journey with digital devices not so long ago. Firstly, I didn’t have enough patience and resigned quickly but I came back to learning because there wasn’t any other option for me. Sometimes I felt alone because I didn’t know how to meet new people. I am really old [laugh] and many of my friends and relatives have died. It annoyed me that I can’t cope with smartphones and Facebook and decided not to give up so fast.*

PLF76

*Using, as you called it, new technologies is now very important if you want to be in touch with others and sustain the feeling of being among the others instead of being alone.*

PLF70

The participants also expressed that the speed of technological developments made them feel left behind and motivated them to improve their digital skills:

*Technology now changes so quickly. I have problems with keeping up with this but I know that I have to if I want to have relations with others. I experienced this feeling of being more alone during the pandemic (COVID-19 pandemic). I want to learn how to use Facebook and Skype but my progress is very poor. I found it too difficult.*

PLF67

*I know that many seniors have problems with using smartphones, computers and digital medical equipment… I am also strongly convinced that they do not feel comfortable with this but have no idea where to search for help. Not everyone has children or grandchildren which would teach you how to cope with a new model of smartphone or Facebook. I am not good in using all of these technologies and really want to learn how to use these but it’s not so easy in practice.*

PLF71

The participants considered successful ageing in relation to the independent use of new technologies.

*I fear that as I get older there will come a time when I won’t be able to keep up with all these technological innovations like smartphones, electronic evidence, digital patient accounts, assistive robots, heart rate monitors, health apps, Facebook and Instagram. I am aware that the pace of change is overwhelming. To age successfully I need to keep up with it.*

PLM88

*I will tell you that I really think that without all these new technologies many people would be lonelier. I experience myself that there is no substitute for real contact with another human being. But on the other hand, although I’m not that old yet [laugh] I already see that a lot is changing around me. So sometimes it’s better to have that online or telephone contact with someone than none at all.*

PLM69

Successful ageing was analyzed by the participants as being related to independence in using new technologies.

*I would like to be able to do everything myself, without the help of others. Then I would feel independent and fully capable. Besides, not everyone needs to know what I’m doing there on the Internet. I don’t like others treating me like a child… This is what worries me, that I will stop keeping up with everything myself. Successful ageing for me means above all maintaining that sense of independence and agency.*

PLM70

*When thinking about the future, I would like to keep up to date with more than just the workings of ever new phones or computers. In fact, for me the most important thing would be to understand all that is changing. I am sure that I already don’t understand well all those emoticons or strange abbreviations in text messages.*

PLM81

*We often say that-people with disabilities. And I guess it’s the case that if someone can’t use a smartphone then they are such a person with a disability. I am such a person. My digital skills are very poor. I hardly know how to use a smartphone. I prefer a dial telephone. I must ask for help even while changing channels on TV.*

PLM73

## 5. Discussion

This study was designed to interpret factors influencing seniors’ successful ageing (including experiences with ageing, ways of organizing life in old age, and expectations towards ageing now and in the future). The most salient findings from this study underlined the importance of an approach focused on an analysis of the given process of ageing in a specific cultural context. The focus on the normative outcomes of success in old age is a very difficult task because of the vast heterogeneity of the ageing process and its social and individual interpretations. Previous research findings were mainly based on the assumption of the need to explore universal standards and norms of successful ageing to draw a general profile of people who reached success in old age [36]. Many studies put efforts into operationalizing universal constructs of successful ageing across heterogeneous categories of older people by offering multicriterial models of its factors and barriers [43,44,45,46,47].

Focusing on the experience of ageing affords a more contextual point of view with a focus on accepting the diversity of personal interpretations of success in old age [48,49]. This study showed that many factors were considered by interviewees as success outcomes. The analysis of the research data leads to an interesting cognitive ambivalence. On the one hand, it should be stated that the category of success is, in a sense, global. When talking about success, we most often refer to certain interpretative patterns learned in the process of socialisation. These cultural clichés dominate the global narrative or discourse on old age and are based on a capitalist ethos. When hearing the word success, participants in the study first mentioned concepts such as work, family, health, and social prestige, before moving on to somewhat negate such a general understanding of the category. When talking about why the definition of successful ageing should not be reduced to these elementary categories, respondents began to refer to different life experiences and the ageing process as a whole. They described their successes, analysing various events that had personal meaning for them, such as a job that gave them freedom of expression or a beautiful garden. Some facilitators may be discussed as embedded in specific cultures and others as more universal standards. Most of these, however, are attributable to the individual experiences of individuals while living with others in society. Accepting life satisfaction evaluation brings us to more specific processes such as transitioning to retirement, using coping strategies in adaptation to negative changes, reaching personal goals, and leading a meaningful life. At this point, it is also worth referring to the general narrative of ageing operating in specific societies [49,50,51]. Successful ageing is a theoretical but also a social construct. In some approaches we see a focus on the idealization of old age, while others emphasize the importance of the losses experienced by older people [47,50]. As this study implies, the most adequate approach is based on an analysis of specific processes/coping strategies that older adults use to achieve their individual goals and lead meaningful lives rather than the chosen outcomes of successful ageing.

Within this study, the mediating factors of successful ageing were supportive environments. This category revealed major differences in the interpretation of the importance of nursing homes and/or care homes for old people (the participants used two different names for these institutions) compared with other research [34,35]. It may bring us to the conclusion that Polish seniors have negative attitudes towards nursing homes, which are assessed very negatively. Living in a nursing home was interpreted by them as a life failure. Many of them defined successful ageing in the future as meeting the one condition of living with the family (children and/or people close to them). When pointing to the importance of being independent while ageing, they also emphasized the necessity of accepting temporary help from people close to oneself. The second assumption was similar to previous research studies. They mentioned multicriterial-defined good health among others factors of successful ageing [51,52]. This research once again focuses our attention on coping strategies in case of health problems, which were discussed as crucial for successful ageing by the participants.

The next research finding focused our attention on the meaning of a supportive environment for successful ageing. Comparing that statement with the previous studies it may be seen that many findings highlight the importance of social relations and social engagement [15,49]. The results are in line with those approaches. The most surprising element was the participants’ focus on independence in using technological advancements as a relevant factor of successful ageing. Within this study, motivation for use of new technologies was primarily related to sustaining social ties, social relations, and social networks. Digital devices were perceived as most beneficial for connecting with others via a communication circle. The older adults have faced and understood specific technological trends and agreed on its importance even if they are not able to keep up with the pace of changes in the level of digital competences. Even in cases where the participant claimed to have problems with using new devices, he or she admitted these to be important for being independent while ageing. In the literature, we find many descriptions of studies on older people’s adaptation to new technologies [29,31,49,50,51,52,53,54,55]. Previously, however, independence in the use of technological advancements has not been directly linked to theories of successful ageing. Within this study, this issue resonated very strongly with the interviewees. Finally, and in summary, it should be stressed that the ageing-process-based approach emphasized the interrelations between gains and losses in old age by introducing a more complex approach that combined the idealistic and negative clichés of successful ageing to advocate for the importance of culturally embedded qualitative studies.

## 6. Research Limitations and Directions for Future Research

The author conducted a relatively small number of interviews. This could be cited as a limitation of the study. The research methodology used, especially the procedure of theoretical data saturation, allows us to conclude that the number of interviewees should not be treated as a limitation of the study in this case. The participants were recruited from different regions of Poland and had different education levels and life experiences. The chosen procedure seemed fit to describe older adults’ experiences, as well as their ways of organizing life in old age and their expectations of old age. One avenue for future research is to conduct a focus-group study among older adults. The second avenue is to conduct a survey on older adults’ attitudes towards ageing on a representative sample in Poland. The third avenue is to perform theoretic studies on the sociological discourse on ageing. The fourth direction may be to undertake an international comparison of stereotypes of ageing in different countries of Europe.

## 7. Conclusions

Within this study, the author used a qualitative and explanatory method to explore factors influencing older adults’ successful ageing. The author’s focus was on how older adults experienced ageing. The second issue was how they had been organizing life in old age. The third point concerned their expectations towards ageing now and in the future. The author conducted semi-structured and open-ended interviews with 13 older adults (60+) in Poland. This logic enabled participants to give rich descriptions of defined problems. The interviews described the individuals’ own experiences, their motivations, perceptions, facilitators, and expectations of successful ageing. Three themes emerged from the coding process, each with their own sub-themes: 1. Life satisfaction (transitioning to retirement, using coping strategies in adaptation to negative changes, reaching personal goals, leading a meaningful life); 2. Supportive environments (being independent but using temporary assistance from relatives and/or people close to oneself, living with family members (e.g., husband/wife, children, grandchildren), having access to health care system); 3. Social integration (social relations, social engagement, independence in using technological advancements). The core category that was derived from these three was social networks, new technologies, and wellbeing. As this study implies, the most adequate approach of studying successful ageing is based on an analysis of specific coping strategies older adults use to achieve their individual goals in order to lead meaningful life while ageing, rather than on the chosen outcomes of successful ageing measured by sets of general factors.

## Figures and Tables

**Table 1:** Socio-demographic characteristics of respondents.

Code *	Age	Gender	Education *	Place of Residence	Health Evaluation *
PLF77	77	Female	Primary	Rural	Average health
PLF76	76	Female	Higher	Urban	Average health
PLF70	70	Female	Secondary	Rural	Average health
PLF72	72	Female	Higher	Urban	Good health
PLF71	71	Female	Secondary	Urban	Average health
PLF65	65	Female	Primary	Rural	Average health
PLF67	67	Female	Primary	Rural	Poor health
PLM88	88	Male	Higher	Rural	Good health
PLM81	81	Male	Secondary	Urban	Good health
PLM69	69	Male	Secondary	Urban	Poor health
PLM73	73	Male	Secondary	Rural	Poor health
PLM70	70	Male	Higher	Urban	Average health

Source: Compiled by the author on the basis of research data; * Code = PL-nationality, F/M-gender, Number-age.

**Table 2:** Themes and subthemes.

Themes	Subthemes
Life satisfaction	Transitioning to retirement
	Using coping strategies in adaptation to negative changes
	Reaching personal goals
	Leading a meaningful life
Supportive environments	Being independent but using temporary assistance from relatives and/or people close to oneself
	Living with family members (e.g., husband/wife, children, grandchildren)
	Having access to health care system
Social integration	Social relations
	Social engagement
	Independence in using technological advancements

## Data Availability

The data supporting reported results can be found at the Institute of Sociological Sciences of The John Paul II Catholic University of Lublin. Availability: on researcher’s request accordingly to the politics of data management at The John Paul II Catholic University of Lublin: alina.betlej@kul.pl.
